# ZEB1: A Critical Regulator of Cell Plasticity, DNA Damage Response, and Therapy Resistance

**DOI:** 10.3389/fmolb.2020.00036

**Published:** 2020-03-19

**Authors:** Stanislav Drápela, Jan Bouchal, Mohit Kumar Jolly, Zoran Culig, Karel Souček

**Affiliations:** ^1^Department of Cytokinetics, Institute of Biophysics of the Czech Academy of Sciences, Brno, Czechia; ^2^International Clinical Research Center, Center for Biomolecular and Cellular Engineering, St. Anne's University Hospital in Brno, Brno, Czechia; ^3^Department of Experimental Biology, Faculty of Science, Masaryk University, Brno, Czechia; ^4^Department of Clinical and Molecular Pathology, Faculty of Medicine and Dentistry, Institute of Molecular and Translational Medicine, Palacky University, Olomouc, Czechia; ^5^Centre for BioSystems Science and Engineering, Indian Institute of Science, Bangalore, India; ^6^Department of Urology, Experimental Urology, Innsbruck Medical University, Innsbruck, Austria

**Keywords:** ZEB1, plasticity, DNA damage response, therapy resistance, EMT-epithelial to mesenchymal transition

## Abstract

The predominant way in which conventional chemotherapy kills rapidly proliferating cancer cells is the induction of DNA damage. However, chemoresistance remains the main obstacle to therapy effectivity. An increasing number of studies suggest that epithelial-to-mesenchymal transition (EMT) represents a critical process affecting the sensitivity of cancer cells to chemotherapy. Zinc finger E-box binding homeobox 1 (ZEB1) is a prime element of a network of transcription factors controlling EMT and has been identified as an important molecule in the regulation of DNA damage, cancer cell differentiation, and metastasis. Recent studies have considered upregulation of ZEB1 as a potential modulator of chemoresistance. It has been hypothesized that cancer cells undergoing EMT acquire unique properties that resemble those of cancer stem cells (CSCs). These stem-like cells manifest enhanced DNA damage response (DDR) and DNA repair capacity, self-renewal, or chemoresistance. In contrast, functional experiments have shown that ZEB1 induces chemoresistance regardless of whether other EMT-related changes occur. ZEB1 has also been identified as an important regulator of DDR by the formation of a ZEB1/p300/PCAF complex and direct interaction with ATM kinase, which has been linked to radioresistance. Moreover, ATM can directly phosphorylate ZEB1 and enhance its stability. Downregulation of ZEB1 has also been shown to reduce the abundance of CHK1, an effector kinase of DDR activated by ATR, and to induce its ubiquitin-dependent degradation. In this perspective, we focus on the role of ZEB1 in the regulation of DDR and describe the mechanisms of ZEB1-dependent chemoresistance.

## Introduction

Cancer is the second leading cause of death worldwide (Wang et al., 2016). Nevertheless, the basis of poor prognosis for cancer patients and the obstacle to a positive clinical outcome is not the primary tumor itself, but cancer cell plasticity, which enables local invasion, dissemination, and distant metastases. Evidence has accumulated that plasticity is driven by the process called epithelial-mesenchymal transition (EMT). Activation of EMT is widely believed to contribute to invasion, metastasis, tumor relapse, and therapy resistance (Zheng et al., 2015). In many epithelial malignancies, EMT is associated with a change in phenotypic features such as loss of cell-cell adhesion and polarity, change from a cobblestone-like shape to an elongated one, and development of a generally more aggressive mesenchymal-like phenotype (Kalluri and Weinberg, 2009). EMT originally observed during embryogenesis (Hay, 1995), is a reversible, evolutionary conserved process that is tightly regulated through the interplay between environmental signals from Wnt, TGF, FGF family members, interleukins, and various EMT-transcription factors (EMT-TFs), including Zinc-finger E-box binding protein 1 (ZEB1), ZEB2, Snail, Slug, and Twist (Kalluri and Weinberg, 2009). All of these processes are fine-tuned by oncogenic and tumor-suppressive microRNAs (miRNA). ZEB1 is a core EMT-TF of the ZEB family and is implicated in cellular plasticity, dissemination, and a dormant-to-proliferative phenotypic switch at the distant site as well as being a determinant of worse clinical prognosis in most human cancers (Zhang et al., 2015; Katsura et al., 2017; Krebs et al., 2017). Furthermore, activation and stabilization of ZEB1 via a miRNA- or ATM-dependent axis contribute to the resistance to various anticancer therapies (Burk et al., 2008; Zhang et al., 2014a). With regards to clinical relevance, ZEB1 expression increases progressively through the different stages of cancer progression, e.g., ZEB1 expression dramatically increases in advanced castration-resistant prostate cancer (CRPC) and PCa metastasis compared to clinically localized prostate cancer (Figiel et al., 2017). Moreover, patients with ZEB1-expressing metastases have shorter overall survival compared to patients with ZEB1-negative tumors (Figiel et al., 2017). In this review, we outline recent studies on the molecular function of ZEB1 in cellular plasticity and metastasis and elucidate its role in DDR and therapy resistance in an EMT-dependent or EMT-independent manner.

## Structure and Regulatory Mechanisms of ZEB1

ZEB1 protein (also known as TCF8), encoded by the ZEB1 gene in humans, is a transcription factor characterized by the presence of two C2H2-type flanking zinc finger clusters, which are responsible for interaction with paired CACCT(G) E-box-like promoter elements on DNA, and a centrally located POU-like homeodomain, not binding DNA (Vandewalle et al., 2009). Additionally, ZEB1 contains Smad- (SID), CtBP- (CID), and p300-P/CAF (CBD) interaction domains that are instrumental in the control of its transcriptional activity (Vandewalle et al., 2009; Zhang Y. et al., 2019). ZEB1 can either downregulate or upregulate the expression of its target genes by epigenetic mechanisms, including DNA methylation or histone modifications and recruitment of different co-suppressors or co-activators through SID, CID, or CBD (Postigo et al., 2003). For instance, ZEB1 can activate transcription of TGF-beta responsive genes through its interaction with co-activators such as Smad, p300, and P/CAF (Postigo et al., 2003; Caramel et al., 2018). Conversely, recruitment of CtBP transcriptional co-repressors (histone deacetylases HDAC1/2) following direct ZEB1 binding onto the *CDH1* gene promoter leads to repression of *CDH1* transcription, resulting in downregulation of E-cadherin protein expression and induction of EMT (Zhang et al., 2015). This dual activity, which fosters the expression of genes encoding components for tight cell junctions, desmosomes or intermediate filaments, is unique for ZEB1/2 transcription factors and crucial for the EMT program (Caramel et al., 2018).

Regulation of ZEB1 expression can be accomplished on different levels by transcriptional or post-transcriptional mechanisms. First, the feedback loop between ZEB1 and the miRNA-200 family is a well-described mechanism of the regulation of cellular plasticity, (de)differentiation, and EMT machinery (Tian et al., 2014; Zhang Y. et al., 2019). Second, ubiquitination by E3 ligase complex Skp1-Pam-Fbxo (Xu et al., 2015) or, conversely, deubiquitination by USP51 enzyme has also been shown to regulate ZEB1 and EMT (Zhou Z. et al., 2017). Expression of ZEB1 is under the control of different positive (TGF-beta, Wnt/beta-catenin, NF-κB, PI3K/Akt, Ras/Erk) as well as negative regulators, including miRNA signaling (Chua et al., 2007; Bullock et al., 2012; Horiguchi et al., 2012; Kahlert et al., 2012; Zhang and Ma, 2012; Zhang Y. et al., 2019). For instance, ZEB1 represents the direct downstream target of Wnt-activated beta-catenin in bone metastasis of lung cancer, resulting in decreased levels of E-cadherin and EMT (Yang et al., 2015). In parallel, TGF-beta induces the mesenchymal phenotype in glioblastoma cells via pSmad2- and ZEB1-dependent signaling, leading to tumor invasion (Joseph et al., 2014). Finally, Han et al. have reported that hepatocyte growth factor increases the invasive potential of prostate cancer cells via the ERK/MAPK-ZEB1 axis (Han et al., 2016). Besides well-known transcription factors, Grainyhead-like 2 (GRHL2) has been described as a potential key player associated with the epithelial phenotype and an important regulator of ZEB1 and EMT. Studies have shown that GRHL2 modulates the expression of E-cadherin and Claudin 4, which are crucial for differentiation and maintenance of cell junctions (Werth et al., 2010). In breast cancer, GRHL2 acts as an EMT suppressor by forming a double-negative feedback loop with the EMT driver ZEB1 via the miR-200 family (Cieply et al., 2012). Similarly, GRHL2 regulates epithelial plasticity along with stemness in pancreatic cancer progression by forming a mutual inhibitory loop with ZEB1 (Nishino et al., 2017). Whereas combined (over)expression of GRHL2 and miR-200s increases E-cadherin levels, inhibits ZEB1 expression and induces MET (Somarelli et al., 2016), GRHL2 knockdown is associated with downregulation of epithelial genes, upregulation of ZEB1 or vimentin, and the onset of EMT (Chung et al., 2019). Hence, the reciprocal repressive relationship between GRHL2 and ZEB1 is considered to be a significant regulator of EMT cell plasticity and chemoresistance (Chung et al., 2019). These regulatory mechanisms make ZEB1 the core downstream target of broad spectra of signaling pathways implicated in various cellular processes, including differentiation, proliferation, plasticity, and survival.

## ZEB1 in Plasticity and Dissemination

Enhanced plasticity of cancer cells is considered an important driving force of tumor progression, allowing continuous adaptations to the demanding conditions in the ever-changing tumor microenvironment. Cellular plasticity is exerted by a reciprocal feedback loop between the EMT driver ZEB1 and the miR-200 family as an inducer of epithelial differentiation (Burk et al., 2008; Gregory et al., 2008; Brabletz and Brabletz, 2010). Within this feedback loop, ZEB1 promotes EMT, plasticity, dissemination, and drug resistance via inhibition of the transcription of miR-200 family members, while miR-200 family members promote MET, differentiation, and drug sensitivity by inhibition of ZEB1 translation (Brabletz, 2012). Thus, this regulatory mechanism was proposed as a molecular “engine” of cellular plasticity and a driving force toward cancer metastasis (Brabletz and Brabletz, 2010). Mathematical modeling of this feedback loop suggests that cells need not necessarily attain just epithelial or mesenchymal states; rather, they can stably acquire a hybrid epithelial/mesenchymal phenotype (Lu et al., 2013). The coupled system of ZEB1, GRHL2, and miR-200 drives the cellular dynamics of epithelial-hybrid-mesenchymal transition (Jolly et al., 2016; Chung et al., 2019). ZEB1 forms two additional indirect feedback loops including epithelial splicing regulatory factor ESRP1 and an enzyme that produces hyaluronic acid, HAS2 (Preca et al., 2015, 2017; Jolly et al., 2018) Thus, ZEB1-mediated feedback loops function as a hub of cellular plasticity during metastasis.

From another point of view, it is increasingly evident that genomic regions that do not encode proteins and are often transcribed into long non-coding RNAs (lncRNAs) represent important regulators of cancer development, dissemination, and aggressiveness (Huarte, 2015). Moreover, lncRNAs can directly interact with proteins and thereby regulate their stability (Huarte, 2015). A recent study has reported a novel lncRNA, namely RP11-138 J23.1 (RP11), as a positive regulator of migration, invasion, and EMT in colorectal carcinoma cells *in vitro* and enhanced liver metastasis *in vivo* (Wu et al., 2019b). Mechanistically, epigenetic upregulation of RP11 (m6A modification) accelerates the degradation of two E3 ligases and thus attenuates proteasomal degradation of ZEB1, resulting in dissemination of CRC cells (Wu et al., 2019b).

Genome-wide screening of ZEB1 targets using TNBC cell line Hs578T revealed more than 2,000 genes that are positively or negatively regulated by this transcription factor. In the context of plasticity, ZEB1 contributed to the regulation of cell polarity via DLG2 and FAT3 proteins, cell-to-cell adhesion via transmembrane protein TENM2 or anchorage-independent growth through interaction with metalloproteinase inhibitor TIMP3 (Maturi et al., 2018). Moreover, strong evidence indicates that ZEB1, but not Snail or Slug, is the master regulator of phenotypic as well as metabolic plasticity of pancreatic cells, affecting cancer cell dissemination and metastasis (Krebs et al., 2017). Moreover, metastasis remains one of the main obstacles in cancer therapy. Hence, effective anti-ZEB1 immunotherapy might serve as a promising tool for the reduction of cancer cell dissemination and metastasis and thus could help to eradicate various types of cancer. Notably, ZEB1 depletion significantly reduces stemness and colonization capacity and locks the cells in the homogeneous epithelial state, limiting cell heterogeneity and plasticity (Krebs et al., 2017). However, the similarities and overlaps in molecular networks mediated by ZEB1 vs. other EMT-inducing transcription factors remain to be identified. Thus, ZEB1 may play context-specific roles in repressing epithelial genes and/or activating genes involved with a mesenchymal phenotype (Watanabe et al., 2019), given its ability to function both as a repressor and as an activator depending on available co-factors (Lehmann et al., 2016).

The detachment of tumor cells from the main tumor bulk and invasion through surrounding stroma is an important step for the development of distant metastasis. A growing body of evidence proves that the stroma plays a major role in the budding of quiescent tumor cells, resulting in dissemination. ZEB1 has been shown to be strongly associated with this complex process, wherein EMT-like stromal cells possessing high ZEB1 levels trigger the tumor-budding phenotype by tumor-stroma crosstalk (Galvan et al., 2015). Strikingly, ZEB1 also governs the inflammatory phenotype in breast cancer cells by regulating the secretion of pro-inflammatory cytokines IL-6 and IL-8 and induction of fibroblasts and growth of myeloid-derived suppressor cells, indicating its key role in the tumor microenvironment and formation of the pre-metastatic niche (Katsura et al., 2017; Carpenter et al., 2018). At the same time, it has been shown that in the post-dissemination events, inflammation orchestrates ZEB1-dependent escape of disseminated tumor cells from dormant to active phenotype and induces EMT-associated metastatic outgrowth, highlighting the importance of ZEB1 in the regulation of cell plasticity (De Cock et al., 2016). Functional studies revealed that ZEB1 overexpression drives melanoma phenotypic plasticity and is sufficient to drive resistance to BRAF and/or MEK inhibitors, whereas ZEB1 inhibition sensitizes naive melanoma cells to BRAF inhibitors, prevents the emergence of resistance, and decreases the viability of resistant cells (Richard et al., 2016). Finally, ZEB1-driven phenotypic plasticity of epithelial pancreatic cancer cells was also observed *in vivo*, where differentiated primary tumor cells underwent dedifferentiation associated with an upregulation of ZEB1 at the invasive front, resulting in liver metastasis (Krebs et al., 2017). Taken together, these findings reveal the crucial role of ZEB1 in the phenotypic plasticity important for the dissemination of cancer cells and the establishment of metastasis in distant sites.

Importantly, ZEB1-mediated mechanisms and feedback loops can also drive an irreversible EMT (Jia et al., 2019). However, the different impacts of reversible vs. irreversible EMT on associated traits such as therapy resistance, immune evasion, and tumor-initiating potential remain to be investigated. Breaking the ZEB1/miR-200 feedback loop has been shown to alter the dynamics of phenotypic plasticity in a cell population and curb metastasis *in vivo*, but the mechanisms involved here are still elusive (Celia-Terrassa et al., 2018).

## Mechanisms of ZEB1-trigged Therapy Resistance

Nowadays, a growing body of evidence implicates intratumoral heterogeneity, EMT, and increased ZEB1 levels as among the main drivers of therapy resistance, exemplified by EMT-induced docetaxel resistance in prostate cancer (Hanrahan et al., 2017), gemcitabine resistance in pancreatic cancer (Wang et al., 2017), and multiple types of resistance within various malignancies (Shibue and Weinberg, 2017; Cui et al., 2018; Zhang et al., 2018; Orellana-Serradell et al., 2019). Besides being a key contributor to the regulation of cancer cell differentiation and metastasis, the potential role of ZEB1 in the modulation of tumor chemoresistance is not yet fully understood.

### Complex Role of ZEB1 in DNA Damage Response and DNA Repair

From previous studies, it is apparent that ZEB1 is required for DNA repair and the clearance of DNA breaks (Zhang et al., 2014b). Mechanistically, ZEB1 knockdown significantly reduces levels of both total and phosphorylated CHK1, a critical effector kinase implicated in DDR and HR-mediated DNA repair, while ZEB1 overexpression acts in the opposite way and promotes clearance of DNA breaks after IR therapy (Zhang et al., 2014b). Previous study showed that chemoresistant tumor cells possess constitutively activated ATM kinase (Svirnovski et al., 2010). This activation is induced and maintained by overexpressed ZEB1 recruiting the transcriptional coactivators p300/PCAF to the *ATM* promoter, which results in the chemoresistance of breast cancer cells (Zhang et al., 2018). Meanwhile, in the positive feedback loop, over-activated ATM stabilizes ZEB1, which acts as a repressor of poly-ubiquitination of endogenous CHK1 via direct interaction with the deubiquitinating enzyme USP7. In contrast, Song et al. claimed that ZEB1 inhibition promotes CHK1 phosphorylation and induces cell cycle arrest in the interphase and thus sensitizes p53-mutated pancreatic cancer cells to the therapy by ATR inhibitor, whereas ZEB1 overexpression attenuates chemotherapy-stimulated CHK1 phosphorylation (Song et al., 2018). This indirect regulation, mediated via interaction of ZEB1 with ATR adaptor protein TopBP1 triggering CHK1 phosphorylation (Song et al., 2018), underlines the pleiotropic and complex role of ZEB1 in the regulation of the response to various anticancer treatments. Importantly, the EMT program has also been shown to be involved in the normal mammary epithelial stem cell state. Morel et al. have shown that ZEB1 is expressed in normal human mammary stem cells and promotes a protective antioxidant program driven by the methionine sulfoxide reductase MSRB3 (Morel et al., 2017). This preemptive program prevents the formation of oncogene-induced DNA damage in stem cells. As a direct consequence, ZEB1 expression precludes the activation of the p53-dependent DNA damage response (DDR) and ensures the maintenance of genomic stability over the course of tumorigenesis. These findings provide a rational explanation for the existence of a subclass of aggressive breast neoplasms exhibiting high ZEB1 expression, a low frequency of p53 mutations, and a subnormal genomic landscape. Given these data, it is evident that ZEB1 plays a significant role in the regulation of DDR and DNA repair machinery, no matter of the p53 status. Considering that DDR is one of the most important signaling pathways in the maintenance of genomic integrity and regulation of cell response to the various anticancer therapies, ZEB1 represents a promising target for combined therapy with DNA-damaging drugs in order to decrease toxicity and undesirable side effects.

### Interplay Between microRNAs, ZEB1, and DNA Damage Response

Cellular plasticity, EMT, and ZEB1 overexpression share one common denominator: microRNAs (miRNAs) (Zhang Y. et al., 2019). Many studies have considered miRNAs as key regulators of EMT through downregulation of EMT-driving transcription factors, including Twist, Snail, and ZEB1/2 (Bullock et al., 2012; Zhang and Ma, 2012; Khanbabaei et al., 2016). Recent studies have highlighted both radio- and chemotherapy when used alone as major factors in cancer cell plasticity, promoting *in vitro* invasion and migration in a ZEB1-dependent manner through the ERK1/2 signaling pathway (Song et al., 2017). For instance, exposure of triple-negative breast cancer cell lines to radiation triggered migration and progression via ATM-driven phosphorylation and stabilization of ZEB1 protein, while its mRNA levels remained unchanged (Lin et al., 2018). Moreover, complete loss or downregulation of different miRNAs was strongly associated with poor prognosis, metastasis, and resistance to various anticancer therapies. Although the mechanism of ZEB1-driven chemoresistance is not yet fully described, miR-203 has been considered an important ZEB1 target with stemness-inhibiting properties and a capability to restore drug sensitivity (Meidhof et al., 2015). Sensitivity to the chemotherapy drug gemcitabine can be restored by targeting the negative feedback loop miR-203-ZEB1 using histone-deacetylase (HDAC) inhibitor mocetinostat (MGCD0103). Mocetinostat interferes with ZEB1, downregulating its mRNA and protein levels, and upregulates tumor-suppressing miR-203, resulting in significantly enhanced sensitivity of pancreatic cancer cells to gemcitabine therapy (Meidhof et al., 2015). Also, miR-205 upregulation enhances radiation response in a prostate cancer cell line as well as in xenograft models by impairment of DDR and DNA repair as a consequence of ZEB1 inhibition (El Bezawy et al., 2019). Moreover, siRNA-mediated silencing of ZEB1 recapitulated the effect of miR-205 re-sensitization, confirming its functional role in radiotherapy of prostate cancer (El Bezawy et al., 2019). Similarly, reconstitution of miR-875-5p, whose expression is strongly down-regulated in prostate cancer clinical samples, led to enhanced radiation response in PCa cell lines and xenografts by disabling EGFR nuclear translocation and upstream signaling of ZEB1-triggered activation of CHK1 and DNA repair machinery (El Bezawy et al., 2017). At the same time, miR-875-5p, counteracts EMT by suppression of EGFR and ZEB1, signaling molecules that are crucial for the preservation of a mesenchymal-like phenotype (El Bezawy et al., 2017). In regard of DDR, the tumor suppressor protein p53 is a crucial molecule in the regulation of the cell cycle (Chen, 2016) and cell differentiation and plasticity (Spike and Wahl, 2011), indicating that p53 deregulation might play a critical role in disease progression, activation of DNA damage, and chemoresistance. Moreover, p53 induces miR-200c transcription, which leads to ZEB1 inhibition and MET (Kim et al., 2011; Schubert and Brabletz, 2011). Thus, an intact p53-ZEB1 feedback loop represents an important regulatory mechanism for epithelial phenotype maintenance, suppression of metastasis, and protection against enhanced chemoresistance. Importantly, two independent studies with MDM2 inhibitors, which both reactivated p53 and downregulated ZEB1, also documented decreased stemness features and glioblastoma aggressiveness (Giacomelli et al., 2017; Her et al., 2018). Such effects of p53 reactivation on ZEB1 may be mediated via activation of microRNAs that p53 can activate such as miR-34, miR-145, and miR-200 (Chang et al., 2011; Siemens et al., 2011; Ren D. et al., 2013).

Further studies have reported other feedback loops whereby the miR-205 and miR-200 family of miRNAs directly target ZEB1 and, conversely, ZEB1 represses the transcription of miR-200 genes (Burk et al., 2008; Gregory et al., 2008). Consistently, irradiation therapy in breast cancer cells results in massive miR-205 downregulation, accompanied by upregulation of ZEB1, which can be completely reversed by inhibition of ATM or direct depletion of ZEB1 (Zhang et al., 2014a). This supports the scenario where ATM stabilizes ZEB1 upon irradiation, which in turn represses its negative regulator miR-205, leading to more robust activation of ZEB1, enhanced DNA repair, and radioresistance. Previous reports have shown that similarly to miRNA-205, miR-200c directly targets ZEB1 (Hurteau et al., 2007) and is crucial for the maintenance of sensitivity to chemotherapy. Since low levels of miR-200c are associated with chemoresistance, high ZEB1 levels, and EMT in advanced breast and ovarian cancer, restoration of its expression is considered as a promising therapeutic approach to overcome limited therapeutic response (Cochrane et al., 2009). In addition to those already mentioned, several other miRNAs including miR-15 (Pouliot et al., 2012), miR-16 (Lezina et al., 2013), miR155 (Pouliot et al., 2012), miR-26a (Lezina et al., 2013), and miR-424 (Xu et al., 2013), were also implicated in the direct targeting of CHK1, including dual targeting by miR-195 (Kim et al., 2018) of both ZEB1 and CHK1 at the same time. The loss of these miRNAs was associated with increased activity of the DNA damage and repair machinery and subsequent resistance to chemotherapy. Besides miRNAs, there is evidence that lncRNAs can also play a significant role in cancer progression, metastasis (Chen et al., 2018), and chemoresistance (Bermudez et al., 2019). For instance, overexpression of lncRNA SBF2-AS1 led to the promotion of temozolomide chemoresistance in glioblastoma cells and tissues via a ZEB1-dependent pathway. ZEB1 was found to directly bind to the *SBF2-AS1* promoter, induce its expression and stimulate double-strand-break DNA repair, thereby increasing chemoresistance spread by exosomes (Zhang Z. et al., 2019). Taken together, these results support the idea that miRNAs regulating ZEB1 expression represent a crucial mechanism controlling DDR, activation of DNA repair, and subsequent chemoresistance.

### EMT: Effector or Bystander of Therapy Resistance?

The epithelial-to-mesenchymal transition refers to the highly conserved trans-differentiation program that culminates in increased tumorigenesis, invasiveness, and metastatic potential and can generate CSCs (Mani et al., 2008; Brabletz et al., 2018) with significantly enhanced potential to stimulate DNA repair and promote therapy resistance (Bao et al., 2006). Emerging evidence indicates that molecular and phenotypic changes during acquired drug resistance are associated with the differentiation state of the tumor, which is likely to reflect EMT and the emergence of chemorefractory cells with stem cell-like features in many cancer types (Voulgari and Pintzas, 2009; Singh and Settleman, 2010). EMT is a multi-dimensional, non-linear process where cells can acquire multiple states along the spectrum of the epithelial-mesenchymal landscape; the association of these states with drug resistance need not be universal but is dependent on cancer, drug, and also the inducer of EMT in that context (Huang et al., 2013).

As one of the major inducers of EMT (Yang and Weinberg, 2008), ZEB1 represents an important molecule that plays a crucial role in tumor progression and metastasis and the expression of which correlates with poor clinical outcome in cancer patients (Shibue and Weinberg, 2017). ZEB1 is also implicated in resistance to various anticancer therapies through both EMT-dependent and EMT-independent mechanisms, depending on specific cancer and treatment type. Thus, it remains unclear whether EMT by itself or specific EMT regulators are the main drivers of therapy resistance. Previous studies demonstrated that highly proliferative non-EMT breast cancer cells were sensitive to chemotherapy, while the emergence of recurrent EMT-derived metastases was associated with resistance to cyclophosphamide *in vivo* (Fischer et al., 2015). Also, miR-200 overexpression results in the switch toward cyclophosphamide sensitivity (Fischer et al., 2015). These results indicate potential relevance of EMT in the chemoresistance, as the main target of miR-200 is ZEB1, a crucial regulator of EMT that is capable of reversing the whole machinery (Bracken et al., 2014). Further studies have discovered various other mechanisms connecting EMT and chemoresistance. For instance, Snail, Slug, and ZEB1 were determined to be inducers of chemoresistance driven by inhibition of p53-mediated apoptosis via ATM and PTEN (Liu et al., 2015). Further, loss of miR-200c led to the induction of Snail and ZEB1, activation of EMT, and abnormal expression of beta-tubulin III (TUBB3), leading to paclitaxel resistance in ovarian cancer cell models (Izutsu et al., 2008). A SIRT6-driven EMT program is sufficient to enhance repair of carboplatin-induced DNA damage by activation of DNA repair enzyme, poly ADP-ribose polymerase (PARP). This mechanism counteracts the cytotoxic effect of this chemotherapeutic agent and results in chemoresistance (Mao et al., 2011).

EMT is also associated with the expression of ATP-binding cassette (ABC) transporters, membrane proteins responsible for pumping xenobiotics out of the cells (Saxena et al., 2011). Indeed, the correlation between EMT, increased ZEB1/2-dependent expression of MDR1 and ABCG2, and resistance to platinum-based drugs was confirmed by the whole transcriptome profiling of ovarian and lung cancer tissues (Zhou Y. et al., 2017; Wu et al., 2019a). Finally, several recent studies demonstrated decreased sensitivity to chemotherapy of primary and metastatic tumor cells in an EMT-dependent manner in both lung and pancreatic cancers (Fischer et al., 2015; Zheng et al., 2015). These reports provide convincing data linking EMT to chemoresistance. Nevertheless, several studies have demonstrated EMT-independent ZEB1-driven chemoresistance. For instance, EMT by itself was considered as an important process contributing to metastasis formation, but not to the limited sensitivity of multiple drug-resistant gastric and breast cancer cell models (Xu et al., 2017). Notably, human lung carcinoma cells resistant to docetaxel possessed significantly increased expression of ZEB1, while other transcriptional factors associated with EMT, including Snail, Twist, and Slug, were not deregulated (Ren J. et al., 2013). Highly expressed ZEB1 was also implicated in several mechanisms leading to chemoresistance to paclitaxel (Sakata et al., 2017) or cisplatin (Cui et al., 2018) in various types of epithelial-like malignancies. Inhibition of ZEB1 in epithelial-like docetaxel-resistant SPC-A1/DTX cells reversed the chemoresistance and significantly enhanced sensitivity to docetaxel (Ren J. et al., 2013). There is increasing evidence that high ZEB1 expression is also one of the significant indicators of poor prognosis in chemoresistant glioblastoma disease (Siebzehnrubl et al., 2013). Experimentally, ZEB1 regulates expression of O-6-methylguanine DNA methyltransferase (MGMT) via a miR-200c- and c-MYB-dependent axis to promote resistance in a presumably EMT-independent context (Siebzehnrubl et al., 2013). Moreover, several studies also report higher sensitivity of mesenchymal-like tumors to neoadjuvant therapy in comparison to epithelial-like subtypes of breast cancer (Carey et al., 2007; Li et al., 2008). Lastly, following previous findings, only ZEB1, but not other transcriptional factors, including Snail or Twist, conferred radioresistance to the breast cancer model MCF7, even without inducing EMT (Zhang et al., 2014b). These results suggest that ZEB1, but not necessarily EMT itself might indeed be the crucial regulator of therapy resistance.

## Conclusion and Future Directions

**Figure 1:**
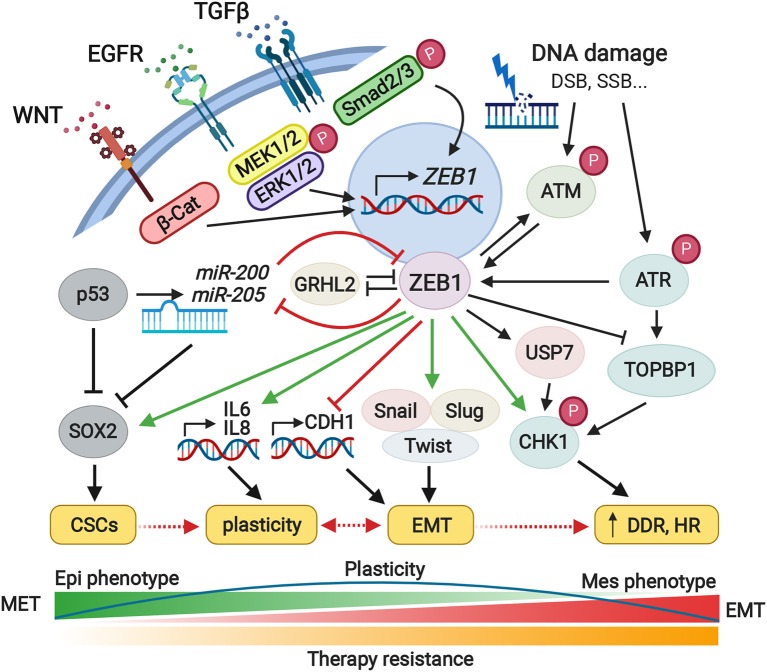
Pleiotropic roles of ZEB1 in the cell plasticity, EMT, and therapy resistance. The ZEB1 represents a core transcriptional factor and central determinant of cell fate which controls fundamental intracellular processes including cell plasticity, EMT, or therapy resistance. Downstream signaling pathways triggered by ZEB1, regulate the activity of the proteins and miRNAs involved in cell differentiation, proliferation, or motility. ZEB1 overexpression is accompanied by overall changeover of the cell phenotype, higher tumorigenic potential, and increased migratory character. ZEB1 also promotes immune escape as well as contributes to the formation of a pre-metastatic niche. Given the tumor heterogeneity, ZEB1 plays an important role in the stemness of cancer cells and increased radio- and chemoresistance. Green and red arrows illustrate major activating or inhibitory effects of ZEB1, respectively. CSCs, cancer stem cells; EMT, epithelial-to-mesenchymal transition; MET, mesenchymal-to-epithelial transition; DDR, DNA damage response; HR, homologous recombination. Created with Biorender.com.

A growing body of publications has considered ZEB1 in normal and cancer cells to be a crucial regulator of fundamental intracellular processes as well a major denominator of plasticity, driving drug adaptation and phenotypic resistance to various types of anticancer therapy. Given the core downstream target of highly conserved pathways implicated in response to DNA damage and repair, proliferation, plasticity, and cell differentiation, ZEB1 plays a pivotal role in the determination of cell fate (Figure 1). Considering its phosphorylation and stabilization by ATM kinase, leading to a limited response to different types of anticancer therapy, combined targeting of ZEB1 with the ATR-CHK1 axis might represent an effective way to overcome these obstacles. Promising results were also obtained with MDM2 inhibitors, which could reactivate p53 tumor suppressor along with downregulating ZEB1 and decreasing stemness features and cancer aggressiveness. An additional possibility for reducing the expression of ZEB1 is inhibition of non-coding circular RNA (circRNA) hsa_circ_0057481, as shown in laryngeal cancer (Fu et al., 2019). Further studies are necessary in order to test clinical applicability. One could also consider inhibition of ZEB1 and EMT by down-regulation of valproic acid, which regulates the *ZEB1* promoter (Zhang S. et al., 2019). Multiple studies have also focused on BET inhibitors in cancer. In this context, it was observed that the DNA endonuclease Mus81, which regulates ZEB1, may be targeted by BET4 inhibitors (Yin et al., 2019). In general, miRNA-based therapeutic options targeting ZEB1 might represent promising tools for targeting ZEB1 but need to be further developed, and delivery methods and therapeutic agent stability should also be investigated with priority.

Despite the association of high ZEB1 expression, EMT, and chemoresistance described in many studies, the role of EMT by itself in therapy resistance is rather controversial. It is not necessarily the epithelial or mesenchymal state that dictates cancer stem-like properties such as drug resistance; instead, they depend on the functions and mechanisms of action of EMT regulators, including ZEB1. Moreover, underlying mechanisms should be investigated for individual context, as the roles of ZEB1 and other transcriptional factors are highly treatment- and cancer type-dependent. The mechanistic links between ZEB1 expression, plasticity, the emergence of CSCs and therapy resistance represent important areas for future investigation. A novel, more specific inhibitors and a better understanding of ZEB1-driven plasticity, inflammation, and vascularization within the tumor and/or pre-metastatic niche microenvironments are inevitably needed to more effectively control resistance to various types of therapies.

## Author Contributions

SD wrote a complete draft and first version of the manuscript. All authors contributed to the manuscript revision, read, and approved final version and contributed to the principle layout of the article.

### Conflict of Interest

The authors declare that the research was conducted in the absence of any commercial or financial relationships that could be construed as a potential conflict of interest.
